# Development of a diagnostic predictive model for determining child stunting in Malawi: a comparative analysis of variable selection approaches

**DOI:** 10.1186/s12874-024-02283-6

**Published:** 2024-08-08

**Authors:** Jonathan Mkungudza, Halima S. Twabi, Samuel O. M. Manda

**Affiliations:** 1 Department of Mathematical Sciences, University of Malawi, Zomba, Malawi; 2 Department of Statistics, University of Pretoria, Pretoria, South Africa

**Keywords:** Child stunting, Area under-receiver operator curve (AUROC), Prediction, MDHS, Model

## Abstract

**Background:**

Childhood stunting is a major indicator of child malnutrition and a focus area of Global Nutrition Targets for 2025 and Sustainable Development Goals. Risk factors for childhood stunting are well studied and well known and could be used in a risk prediction model for assessing whether a child is stunted or not. However, the selection of child stunting predictor variables is a critical step in the development and performance of any such prediction model. This paper compares the performance of child stunting diagnostic predictive models based on predictor variables selected using a set of variable selection methods.

**Methods:**

Firstly, we conducted a subjective review of the literature to identify determinants of child stunting in Sub-Saharan Africa. Secondly, a multivariate logistic regression model of child stunting was fitted using the identified predictors on stunting data among children aged 0–59 months in the Malawi Demographic Health Survey (MDHS 2015–16) data. Thirdly, several reduced multivariable logistic regression models were fitted depending on the predictor variables selected using seven variable selection algorithms, namely backward, forward, stepwise, random forest, Least Absolute Shrinkage and Selection Operator (LASSO), and judgmental. Lastly, for each reduced model, a diagnostic predictive model for the childhood stunting risk score, defined as the child propensity score based on derived coefficients, was calculated for each child. The prediction risk models were assessed using discrimination measures, including area under-receiver operator curve (AUROC), sensitivity and specificity.

**Results:**

The review identified 68 predictor variables of child stunting, of which 27 were available in the MDHS 2016–16 data. The common risk factors selected by all the variable selection models include household wealth index, age of the child, household size, type of birth (singleton/multiple births), and birth weight. The best cut-off point on the child stunting risk prediction model was 0.37 based on risk factors determined by the judgmental variable selection method. The model’s accuracy was estimated with an AUROC value of 64% (95% CI: 60%-67%) in the test data. For children residing in urban areas, the corresponding AUROC was AUC = 67% (95% CI: 58–76%), as opposed to those in rural areas, AUC = 63% (95% CI: 59–67%).

**Conclusion:**

The derived child stunting diagnostic prediction model could be useful as a first screening tool to identify children more likely to be stunted. The identified children could then receive necessary nutritional interventions.

**Supplementary Information:**

The online version contains supplementary material available at 10.1186/s12874-024-02283-6.

## Background

Child malnutrition remains a public health burden in most developing countries, particularly in Sub-Saharan Africa (SSA) and South Asia. An important measurement of childhood malnutrition is stunting, which is defined as poor linear growth, indicated by height for age less than -2 standard deviations from the World Health Organization (WHO) 2006 child growth standards median [1] SSA has the highest burden of stunted children with over one-third of children in 2019 [2]. Worldwide, over 3.1 million children die annually, either directly or indirectly, because of malnutrition and many more suffer from impaired growth as a result of malnutrition [3]. The problem of childhood stunting is a focus of some global initiatives including the Global Nutrition Targets for 2025 and the Sustainable Development Goals for Zero Hunger [4]. The World Health Organization (WHO) considers stunting a public health problem when the prevalence of stunting among children under five years of age is greater than 20% [5]. The prevalence of child stunting was 39% in 2019, thus placing Malawi as a country with a huge health problem regarding child stunting [6].

Childhood stunting affects child morbidity and mortality adversely resulting in poor childhood development and educational performance and increases the risks of infections, which may contribute negatively to adult health and economic productivity [7, 8]. Timely and accurate detection of children who are most likely to be stunted may ensure the prevention of detrimental health outcomes. It may help in delivering tailor-made interventions for the optimum use of available resources. Achieving this goal necessitates the development of diagnostic or clinical prediction models, also known as clinical prediction rules, prognostic models, or risk scores, depending on the study’s aim and design. Prognostic prediction models are designed to calculate the probabilities of specific patient outcomes over time by considering a variety of clinical and non-clinical factors. Diagnostic prediction models estimate an individual’s probability of having a specific health condition (often a disease) at a given time [9, 10]. The aim is to tailor policy decisions to meet the needs of individuals [11–13].

A risk prediction model is a mathematical tool that combines different predictor variables to estimate the probability of occurrence of an outcome of interest [14, 15]. The determination of specific parameter values for the predictor variables, derived from data the model has not seen (the training data), is crucial for calculating a risk score. For prediction models, the choice of the modelling approach is guided by the nature of the predicted outcome. A linear regression method is employed when dealing with continuous outcomes. However, when the outcome of interest is binary, as in the case of predicting stunting status (stunted or not), a logistic regression model is employed. This logistic regression model effectively assesses the likelihood of a binary outcome occurring based on the given set of predictor variables. A Cox proportional hazards regression model is used for a scenario where the outcome is related to the timing of an event, like the occurrence of a particular event over time enabling predictions about the timing of particular events of interest. For count data, a Poisson or negative binomial model has been proposed [16].

Regression modelling faces challenges like overfitting and non-convergence, mainly when dealing with large datasets containing numerous variables. Variable selection methods have been developed to address these issues to identify a concise set of influential predictors from a larger pool. Standard methods, including backward elimination, forward selection, and stepwise selection, are preferred due to their straightforward algorithms [17]. Recent advancements have introduced penalised selection procedures such as the least absolute shrinkage and selection operator (LASSO) and adaptive LASSO. These have gained popularity for enhancing model prediction and inference by simplifying the model. Moreover, tree-based methods like Random Forests and Boruta have been employed to improve accuracy in selecting the most relevant variables from a vast pool, addressing limitations observed in test-based variable selection procedures [18]. Alternative approaches, such as the firefly algorithm, are employed for counting and skewed outcomes using Poisson or Negative Binomial and gamma distribution, respectively [16, 19]. Various simulation studies have demonstrated the effectiveness of these variable selection methods under different conditions [18, 20, 21]. Applying these methodologies to a survey-based study in Malawi, this paper aims to use the variable selection approaches to identify the best predictors that can be used to develop and validate a child stunting diagnostic prediction model.

Determinants of stunting are well-known and studied. However, few studies in Sub-Saharan Africa have developed and validated risk-predictive models to identify children likely to be stunted [22]. One such study was conducted by Hasegawa et al. [23], who developed a screening tool to predict malnutrition among young children in Zambia [24]. The study, however, used data collected from a restricted area (one health facility) from a rural location, limiting the model’s generalizability. The Lives Saved Tool is another predictive model used to estimate the impact of specified changes in key interventions on stunting among children under five years [25]. The Lives tool falls short of predicting which children are stunted or not. Hanieh et al. [25], also developed and externally validated an early life predictive model to predict the risk of stunting in preschool children in Vietnam at three years of age. This stunting prediction model has limitations as it was developed and validated in a rural setting, leaving out urban locations and other areas. Also, the prediction was not based on all ages below five years.

Malawi is a developing country and suffers a shortage of skilled community health workers (HW) and resources to timely detect stunting among children. Thus, modelling techniques emerge as valuable tools that could aid community health workers in promptly identifying children at risk of stunting. This would inform the formulation of proper policies to overcome the challenge of child stunting. Numerous studies have previously been conducted to identify predictors of stunting in Sab-Saharan Africa, specifically in Malawi however, very few studies have been conducted to combine these predictors into a mathematical model to predict stunting. This study aimed to derive and validate a diagnostic risk (score) model that could help identify children who are stunted in Malawi using a nationally representative cross-sectional sample.

## Variable selection methods

Using variable selection algorithms, namely forward selection, backward elimination, stepwise selection; LASSO; random forest, and judgmental selection, different sets of variables were selected from the list of candidate predictors to identify relevant predictors of stunting. The research used more than one variable selection method to compare the predictive ability of each model fitted using the different sets of variables selected by different methods. The following sections provide a brief description of the methods used.

### Least Absolute Shrinkage and Selection Operator (LASSO)

LASSO is a linear regression analysis method that reduces both the sum of squares of errors and the sum of the absolute values of regression coefficients. The regression coefficient $\widehat{\beta ,}$ is determined by minimizing the following formula:

$$\mbox{min} \boldsymbol{\beta} \left\{\frac{1}{2} {\sum }_{i=1}^{n}\left({y}_{i}-{X}_{i}^{T}\boldsymbol{\beta} \right)+\lambda {\sum }_{j=1}^{p}\left|{\beta }_{j}\right|\right\}$$

**β** represents the vector of regression coefficients.

${y}_{i}$ is the observed response for the i-th observation.

${X}_{i}$ is the vector of predictor variables for the i-th observation.

λ is the regularization parameter that controls the value of the penalty term.

LASSO is an algorithm that has a built-in variable selection method. The use of LASSO as a feature selection technique can be seen from the fact that decreasing the values of $\lambda$ in the equation below leads to shrinkage of regression coefficients and some of these even become zero.

### Random forest

Boruta [26], is a non-linear Random Forest (RF)-based variable selection method. It performs feature selection by identifying important features from many potential predictors. It is based on the random forest process. Random shuffling of the original variables creates shadow features. Variable importance for each shadow feature is computed and the highest score becomes the threshold for selection. The shadow features are permuted at random for each new RF iteration. A hit is defined as the number of times a variable has a higher relevance score than maximal importance of random variables. Using the properties of binomial distribution with probability of success *p* = 0.5, it is easy to argue that for N number of RF shuffles, the expected number of hits is E(N) = 0.5N, while the standard deviation is 0.25N. If the importance of the original feature is higher than the threshold, the variable is kept in the model, otherwise, it is discarded. The method comes to a halt when only the most important variables remain in the test or when the maximum number of iterations with some uncertain features has been reached. The Boruta algorithm was implemented in the random forest’s R package (Boruta).

### Forward, backward and stepwise selection methods

Forward selection starts with an empty model and adds variables one at a time. At each step, the algorithm evaluates all potential predictors and selects the one that results in the greatest increase in a chosen criterion, Akaike Information Criterion (AIC). Backward elimination begins with a model that incorporates all potential predictor variables. At each step, the method evaluates the impact of removing each variable and selects the one whose removal results in the least deterioration in the chosen criterion. Stepwise selection alternates between forward and backward steps. At each step, it evaluates both adding and removing variables and chooses the action that optimizes the selected criterion.

The AIC is used as a stopping rule for the forward, backward, and stepwise variable selection methods. AIC is given as;

$$AIC=2K-2\text{ln}L\left(\widehat{\beta }\right)$$

Where K is the number of estimated parameters in the candidate model and $L\left(\widehat{\beta }\right)$ is the estimate from the log-likelihood function. AIC quantifies the comparative information content of a model by utilizing maximum likelihood estimates and counting the number of parameters involved in the model, as indicated in the above-mentioned formula. It is employed to assess and distinguish various potential models, helping in the identification of the best-fit model that is consistent with the given data. It is also used as a stopping rule in variable selection methods. The model giving the smallest AIC over the set of models considered is selected as the best model.

### Judgement variable selection method

Numerous methods for variable selection have been proposed; however, there is no consensus on a single approach that consistently performs well under all circumstances. Therefore, for each dataset, the technique for variable selection should be carefully chosen [27]. In statistical analysis, prior knowledge derived from scientific literature is considered the primary basis for determining the inclusion or exclusion of covariates. However, such information may not always be accessible for all research questions [28]. The judgement variable selection method relies on field expertise acquired through reviewing relevant literature and consulting with experts.

## Data source

The study used data on under-five children extracted from the 2015–16 Malawi Demographic and Health Survey (MDHS). The MDHS is a nationally representative survey conducted by the National Statistical Office in collaboration with the demographic health survey program funded by the United States Agency for International Development (USAID). The MDHS collected up-to-date information on mothers’ demographic and health information on child nutrition. The MDHS collected anthropometric data for the under-five children in selected households. The study analyzed data on 5149 children who had a stunting outcome. Details of the sampling procedure for the MDHS can be obtained in the 2015–16 MDHS report [29].

### Outcome and predictor variables

The outcome variable of this study was stunting and was calculated based on the anthropometric indicator (height-for-age) among under-five children. The height-for-age z-score of the children was calculated using growth standards published by the WHO in 2006 [1]. The height-for-age z-score is a metric used to assess linear growth retardation and cumulative growth deficits in children. Children with height-for-age Z-score below minus two standard deviations (-2SD) from the median of the WHO reference population were considered stunted [30]. To identify determinants of childhood stunting, we conducted a subject review of the available literature PubMed and Google scholar databases was conducted for relevant articles between August to December 2021 to see what has been studied and found about the determinants of childhood stunting in sub-Saharan Africa. Several searches were performed with the search terms “Determinants of stunting AND Africa” or “Risk factors of stunting AND Africa” or “Predictors of stunting AND Africa”. All duplicate articles and those not done in Sub-Saharan Africa were eliminated from the results.

### Statistical methods

All model development and analysis were performed with R software (Version 3.6.2, R Foundation). The data were randomly partitioned into a training set (80%) and a testing set (20%). Six multivariate logistic regressions were trained using the sets of variables selected by the six variable selection algorithms on the training set (80%), and the models were validated on the remaining test set (20%). Another logistic regression model was also fitted based on the risk factors commonly selected by all variable selection algorithms. These models were compared for their discriminative ability and predictive performance. The R package glmnet statistical software (R Foundation) was used to perform the logistic regression. The probability cut-off points (discriminative value) that define positive and negative test results were estimated using the SpEqualSe criterion implemented in the OptimalCutpoints package in R using the training data set (80%). SpEqualSe criterion is the method for computing the optimal cut-off point, which minimizes the absolute deference of sensitivity and specificity [31]. Most often, research studies utilize a default cut point of 0.5 [23, 32, 33]. For comparison purposes, For the sake of comparison, the study also used the cut-off point of 0.5 derived from the Bayes rule. The estimated probability cut-off points were then utilized to derive several performance measures, including the Area under the Operating Curve (AUROC). The AUROC was employed as a metric for assessing the model’s discriminative ability. In the formation of the receiver operating characteristic (ROC) curve at various thresholds, the “true positive rate” (TPR) was plotted against the “false positive rate.” AUC values range from 0.50 to 1. A greater AUC indicates a higher predictive capacity. A model with an AUC of 1.0 is a perfect discriminator, 0.90 to 0.99 is considered excellent, 0.80 to 0.89 is good, 0.70 to 0.79 is fair, and 0.51 to 0.69 is considered poor. Sensitivity was calculated using the following formula: Sensitivity = True Positives / (True Positives + False Negatives). Specificity was calculated using the following formula: Specificity = True Negatives / (True Negatives + False Positives).

## Results

### Distribution of stunting in the data set

Overall, the prevalence of child stunting was 35.6% (95% CI: 34.2% -36.9%). In the training set, the prevalence was 35.9% (34.4%—37.4%) and 34.5% (95% CI: 31.5%—37.6%) in the testing data set.

### Potential predictor variables

The study reviewed 28 papers and identified 68 potential predictors of child stunting from various sources, including [30, 34, 35] particularly in the context of Sub-Saharan Africa, with 27 found in the Malawi Demographic and Health Survey (MDHS). Additional sources are provided in the Appendix section. The predictive variables for child stunting identified in the MDHS included demographic factors; mother’s education level, ethnicity, child’s age, type of residence, child’s gender, household head’s age, marital status, number of under-five children in the household, mother’s age, mother’s body mass index, family size, religion, and region. Economic factors; household wealth index and mother’s occupation. Obstetric and child morbidity variables; childbirth weight, birth order, mode of delivery, diarrhoea episodes, child anaemia level, preceding birth interval, place of delivery, number of births, type of delivery assistance, cough or fever episodes, and distance to the nearest health facility.

### Predictor selection

**Fig. 1:**
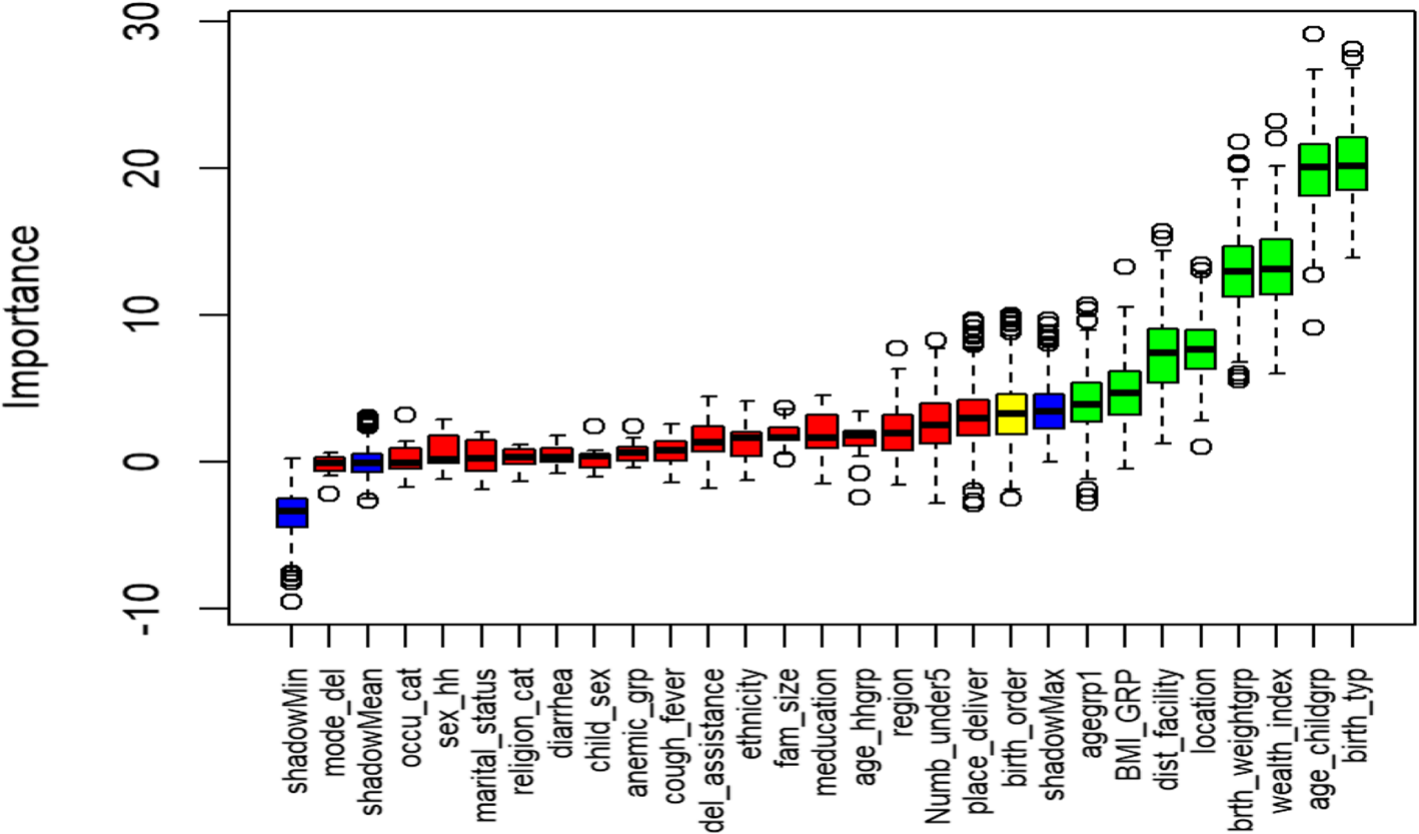
Selected variables: Random Forest, Boruta. *(birth_typ* = *birth type, age_childgrp* = *age of child, wealth_index* = *wealth index, brth_weightgrp* = *child birth weight, location* = *location, dist_facility* = *distance to a health facility, birth_order* = *birth order, age_hhgrp* = *age of household head, BMI_GRP* = *body mass index, fam_size* = *family size, agegrp1* = *mother’s age, place_deliver* = *place of delivery, Numb_under5* = *number of under five children, meducation* = *mother’s education level, del_assistance* = *delivery assistance, marital_status* = *marital status, child_sex* = *sex of child, sex_hh* = *sex of household head, religion_cat* = *religion of mother, anemic_grp* = *anemia, mode_del* = *mode of delivery, occu_cat* = *mother’s occupation, cough_fever* = *cough/fever)*

Boruta (Random Forest) selected 10 variables, which are; the type of birth (single/multiple), age of the child, wealth index, birth weight of the child, location (rural/urban), distance to health facility, age of household head, birth order, body mass index of the mother and size of the household (see Fig. 1). In Fig. 1, the confirmed important variables are those shown in green and those in red represent variables that are not important.

The variables selected by LASSO included location, wealth index, maternal age, age of household head, age of the child, household size, body mass index of the mother, distance to a health facility, number of under-five children, religion, maternal education, type of birth, birth order of the child, region, diarrhoea, maternal occupation, anaemia, delivery assistance, sex of the child, and sex of household head, (see Table S1).

The risk factors selected by backward, forward, and stepwise variable selection algorithms are indicated in Table 1.

**Table 1:** Variables selected by automated methods

Backward	Forward	Stepwise
Age of child	Age of child	Age of child
Type of birth	Birth weight	Birth weight
Wealth index	Type of birth	Type of birth
Body mass index of the mother	Wealth index	Wealth index
Maternal education	Body mass index of the mother	Body mass index of the mother
Sex of the child	Ethnicity	Ethnicity
Number of under-five children	Sex of the child	Sex of the child
Diarrhoea	maternal occupation	maternal occupation
Distance to a health facility	Distance to a health facility	Distance to a health facility
Household size	Location	Location
Delivery assistance	Diarrhoea	Diarrhoea
Age of household head	Number of under-five children	Number of under-five children
Marital status		

**Fig. 2:**
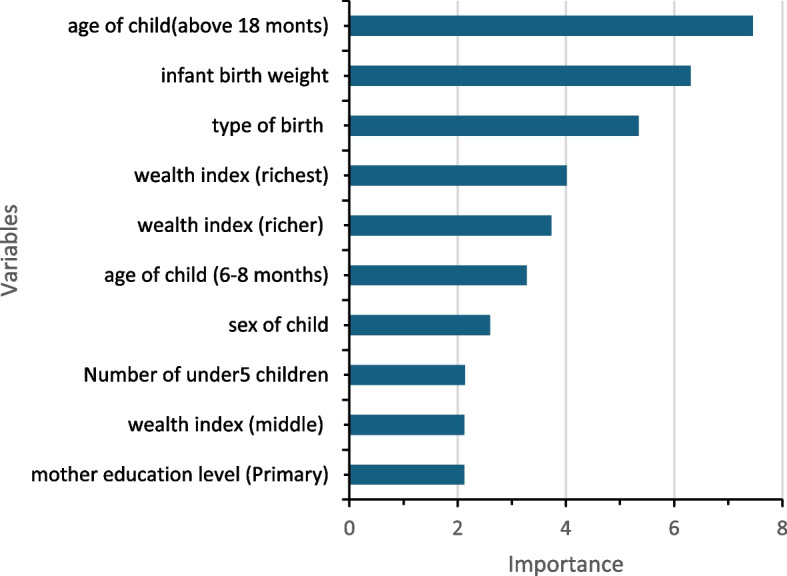
Variable importance plot for the best model (Judgement model)

All variable selection methods selected five common factors: household wealth index, child age, household size, type of birth (singleton/multiple births), and birth weight. The top five most important variables according to variable importance ranking in the best model were the age of a child, birth weight of a child, type of birth, wealth index, and sex of the child (see Fig. 2).

### Model evaluation and performance

The best cut-off point was at probability (stunting) equal to 0.37. The model based on risk factors determined by judgment outperformed the other models AUC = 64% (95% CI: 60%-67%) and 65% (95% CI: 64%-67%) in the testing set and training set, respectively (Tables 2 and 3). Based on the risk factors identified commonly by all variable selection methods, the predictive performance was 62% (95% CI: 59.0%-66.0%). The sensitivity indicates that 61% of the children, who were stunted, were correctly classified as being stunted and the specificity of 60% indicates that children who were not stunted were correctly classified as not being stunted. PPV for all the models were high for the cutoff points of 36% and 37% ranging between 0.71 to 0.74 (Tables 2 and 3). In the study, the performance estimates based on the cutoff point of 0.5 for the test data do well in terms of specificity but all have some poor sensitivity and low PPV’s ranging between 0.40 to 0.57 (see Table 4). The estimated cutoff points for each model seek an equilibrium between sensitivity and specificity. This is important for this study as the interest is in detecting a stunted child than finding a non-stunted child. Hence, more attention is on the sensitivity than the specificity.

**Table 2:** Model performance measures using estimated cutoff points on the training set

	**Cut point**	**AUC (95% CI)**	**Sensitivity**	**Specificity**	**Misclassification error**	**Accuracy**	**NPV**	**PPV**
Model 1	0.36	0.66(0.64–0.68)	0.62	0.62	0.39	0.61	**0.74(0.72–0.76)**	**0.45(0.43–0.47)**
Model 2	0.36	0.66(0.65–0.68)	0.62	0.62	0.38	0.62	**0.74(0.72–0.76)**	**0.45(0.43–0.47)**
Model 3	0.36	0.66(0.65–0.68)	0.62	0.62	0.38	0.62	**0.74(0.72–0.76)**	**0.45(0.43–0.47)**
Model 4	0.37	0.65(0.64–0.67)	0.61	0.61	0.39	0.61	**0.73(0.72–0.76)**	**0.42(0.40–0.44)**
Model 5	0.37	0.67(0.65–0.69)	0.63	0.63	0.37	0.63	**0.73(0.71–0.75)**	**0.44(0.42–0.47)**
Model 6	0.37	0.65(0.64–0.67)	0.61	0.60	0.39	0.61	**0.74(0.72–0.75)**	**0.45(0.42–0.47)**
Model 7	0.37	0.64(0.62–0.66)	0.60	0.61	0.39	0.61	**0.72 (0.70–0.75)**	**0.42 (0.39–0.45)**

Model 1 was constructed using the variables selected by backward variable selection algorithms. Model 2 was constructed using the variables selected by forward variable selection algorithms. Model 3 was constructed using the variables selected by stepwise variable selection algorithms. Model 4 was constructed using the variables selected by random forest variable selection algorithms. Model 5 was constructed using the variables selected by LASSO variable selection algorithms. Model 6 was constructed using the variables selected by judgment. Model 7 was constructed using the variables that were common to the 6 models

*PPV* Positive Predictive Value, *NPV* Negative Predictive Value

**Table 3:** Model performance measures using estimated cutoff points on the test data

	**Cut-off point**	**AUC (95% CI)**	**Sensitivity**	**Specificity**	**Misclassification error**	**Accuracy**	**PPV**	**NPV**
Model 1	0.36	0.63(0.59–0.66)	0.57	0.59	0.42	0.58	**0.73 (0.67–0.78)**	**0.37 (0.33–0.40)**
Model 2	0.36	0.62(0.59–0.66)	0.57	0.59	0.42	0.58	**0.74 (0.69–0.78)**	**0.40 (0.36–0.43)**
Model 3	0.36	0.62(0.59–0.66)	0.57	0.59	0.42	0.58	**0.74 (0.69–0.78)**	**0.40 (0.36–0.43)**
Model 4	0.37	0.62(0.59–0.66)	0.58	0.61	0.40	0.60	**0.74 (0.69–0.78)**	**0.39 (0.36–0.043)**
Model 5	0.37	0.62(0.59–0.67)	0.54	0.62	0.41	0.59	**0.72 (0.68–0.76)**	**0.40 (0.37–0.45)**
Model 6	0.37	0.64(0.60–0.67)	0.61	0.60	0.40	0.60	**0.74 (0.71–0.78)**	**0.44 (0.40–0.48)**
Model 7	0.37	0.62(0.59–0.66)	0.59	0.59	0.41	0.59	**0.71 (0.69–0.76)**	**0.41(.038–0.47)**

Model 1 was constructed using the variables selected by backward variable selection algorithms. Model 2 was constructed using the variables selected by forward variable selection algorithms. Model 3 was constructed using the variables selected by stepwise variable selection algorithms. Model 4 was constructed using the variables selected by random forest variable selection algorithms. Model 5 was constructed using the variables selected by LASSO variable selection algorithms. Model 6 was constructed using the variables selected by judgment. Model 7 was constructed using the variables that were common to the 6 models

*PPV* Positive Predictive Value, *NPV* Negative Predictive Value

**Table 4:** Model performance measures using a cutoff point of 0.5 on test data

	Cut point	AUC (95% CI)	Sensitivity	Specificity	Misclassification error	Accuracy	PPV	NPV
Model 1	0.5	0.63(0.59–0.66)	0.34	0.81	0.35	0.65	0.40 (0.35–0.45)	0.68 (0.65–0.72)
Model 2	0.5	0.59(0.56 -0.63)	0.27	0.83	0.37	0.64	0.41 (0.34–0.48)	0.67 (0.64–0.70)
Model 3	0.5	0.59(0.56 -0.63)	0.27	0.83	0.37	0.64	0.41 (0.34–0.48)	0.67 (0.64–0.70)
Model 4	0.5	0.57(0.53 -0.61)	0.25	0.8	0.39	0.61	0.45 (0.38–0.53)	0.68 (0.64–0.71)
Model 5	0.5	0.62(0.59–0.66)	0.18	0.91	0.34	0.66	0.46 (0.38–0.54)	0.68 (0.64–0.71)
Model 6	0.5	0.64(0.60–0.67)	0.17	0.93	0.33	0.67	0.57 (0.47–0.66)	0.68 (0.65–0.71)
Model 7	0.5	0.63(0.59–0.66)	0.15	0.93	0.34	0.66	0.48 (0.39–0.55)	0.68(0.65–0.70)

Model 1 was constructed using the variables selected by backward variable selection algorithms. Model 2 was constructed using the variables selected by forward variable selection algorithms. Model 3 was constructed using the variables selected by stepwise variable selection algorithms. Model 4 was constructed using the variables selected by random forest variable selection algorithms. Model 5 was constructed using the variables selected by LASSO variable selection algorithms. Model 6 was constructed using the variables selected by judgement. Model 7 was constructed using the variables that were common to the 6 models

*PPV* Positive Predictive Value, *NPV* Negative Predictive Value

**Fig. 3:**
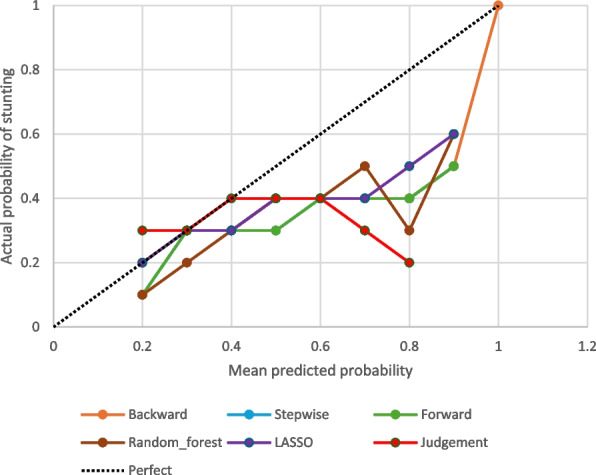
Calibration plots for the fitted models

Calibration plots illustrating the analysis results for each model are presented in Fig. 3. The calibration performance varied across models, with some models demonstrating good calibration at specific probability ranges, as indicated by the calibration slopes and gradients, while others showed poor discrimination. For instance, the judgment model showed good calibration for predicted probabilities between 0.3 and 0.4, whereas the LASSO model performed well for probabilities ranging from 0.2 to 0.3.The selected model has provided a predictive tool that displays a good ability to discriminate between stunted children and those not stunted, particularly in children residing in urban areas (AUC = 67% (95% CI: 58–76%) in children living in urban versus AUC = 63% (95% CI: 59–67) in children living in rural areas) (Table 5).

**Table 5:** Performance of the selected model after adjusting for sex and residence

	Sex of a child		Residence	
	Female	Male	Urban	Rural
AUC (95% CI)	0.64 (0.59–0.70)	0.63 (0.58 -0.68)	0.67(0.58–0.76)	0.63 (0.59–0.67)
Sensitivity (%)	0.86	0.79	0.62	0.7
Specificity (%)	0.26	0.27	0.59	0.45

**Fig. 4:**
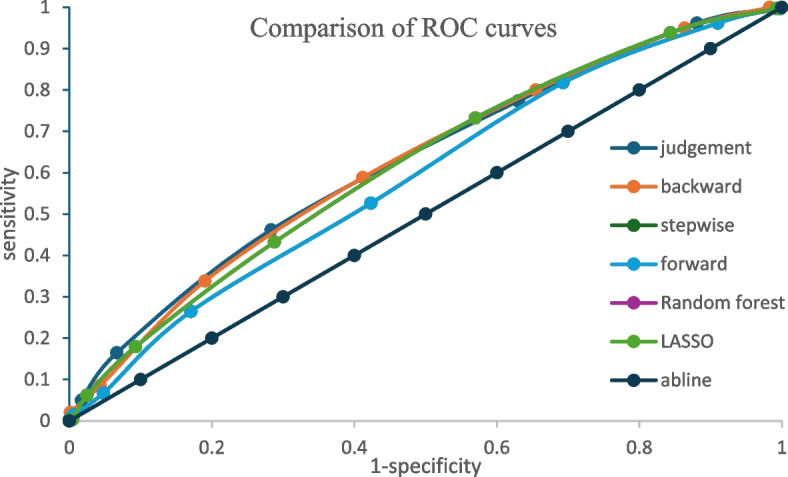
Comparing discrimination of the models fitted using variables selected by the different methods

Figure 4 presents the receiver operating characteristic curve for the models. The findings of this study show that the six prediction models have a better discrimination ability compared to a random classifier as indicated by the ROC curves in Fig. 3.

## Discussion

The study set out to develop and validate a child stunting prediction score based on the best predictive model in Malawi. The study aimed to develop a model that could accurately classify a child as stunting using important factors available in the data. The model was based on predictor variables obtained from fitting a multivariate logistic regression model to child stunting using data from the 2015–16 Malawi Demographic and Health Survey (MDHS). Using six variable selection methods, namely backwards, forward, stepwise, Boruta based on the random forest, LASSO, and judgment, we identified nine easily measured key predictors of child stunting.

The best-performing model was based on the predictors selected using the judgment method, and these included the age of the child, the weight of the child at birth, type of birth, sex of the child, wealth index category of the household, number of under-five children in the household, location, and maternal education. The discriminative ability of our model was different for the type of residence of a child. Our model has shown better predictive performance for children residing in urban areas than those living in rural areas. The discriminative performance of our model was better than that of Hasegawa et al. [23] (AUC = 0.67, 95% CI: 0.64, 0.69), which used data from one health facility. The predictors of stunting identified by our best-performing model were consistent with existing knowledge of determinants of stunting, such as child demographics and wealth indicators.

Our study demonstrated that the best-performing predictive model was based on variables selected using the judgmental variable selection method. In contrast, other studies focusing on childhood stunting in Bangladesh and Rwanda have reported that the gradient-boosting classifier, followed by the random forest algorithm, achieved the lowest classification error in predicting stunting [22, 36, 37]. Some studies have also used the logistic and probit models for stunting prediction due to their common usage in predicting binary outcomes [38]. Other machine learning methods, such as Elastic net, regularized random forests and gradient-boosted feature selection, have been shown as the most effective methods for predicting stunting and other binary outcomes [39]. These help remove less important variables from large datasets with numerous variables for the derived models.

The performance of our final diagnostic risk model for child stunting was comparatively lower than that reported in other studies that developed risk prediction models for child stunting [40, 41]. According to the literature, numerous factors are associated with stunting; however, our study was limited to variables available in the dataset. A similar study by Haque et al. [42], which utilized a randomized cluster design to predict child stunting, also found that the predictive performance of their models, including logistic and probit regression and non-parametric decision trees, was suboptimal. They attributed this to the absence of crucial factors such as environmental and biological variables in their data [42]. Of note, our study calculated the metrics for assessing the performance of the predictive model using cut-points derived based on the SpEqualSe method implemented in the OptimalCutpoins package in R [31]. This method is based on the principles of balancing sensitivity and specificity with the assumption that the costs of false positives and false negatives are equal. The study acknowledges that other methods are used in choosing the probability cut points- such as Youden’s Index (J). The Youden’s Index (J) is defined as the sum of sensitivity and specificity minus one (J_c_ = SE_c_ + SP_c_- 1). Xu et al., [43] used Youden’s Index to determine the optimal value for predicting Acute Kidney Injury (AKI) in their model [44]. Both methods are data-driven and produce similar estimates. However, using the data-driven methods of choosing optimal cut points in studies with small sample sizes may identify inaccurate optimal cutoff points and overstate accuracy estimates [45]. Using pre-specified cutoff points when available would improve the validity of a classification model [46]. These pre-specified cutoff points are the ones that are predetermined by using previous studies, and they are always not available. Our study used a big sample size, which might have avoided what Bhandari et al., [45] observed. By using the model’s optimal cutoff point, the research assesses the performance of a model at one point, but it is important to assess the performance of a model at different cutoff points.

The strength of our approach is that from a large range of candidate predictors from nationally representative data (MDHS), the research was able to identify a small set of key variables routinely measured at the primary healthcare level in many countries or that could be easily obtained. Even though factors affecting stunting that have been reported in the literature vary by many attributes such as type of study, region, sample size, and the ones mentioned above, considerable key findings have emerged that provide support for predictive variables that our model has identified.

The study had some limitations. Firstly, some variables were not captured in the MDHS, and some had missing values, as such, they were not used in developing our predictive model which may have affected the accuracy of our diagnostic predictive model. In addition, the study did not consider the clustering and weighting of the MDHS data, which may have affected the estimated probabilities of being stunted by not being representative. Although the majority of the variables employed were binary, maintaining a linear relationship between the log odds of stunting and the binary indicator points, it is important to note the potential for non-linear relationships with the log odds of stunting for certain categorical variables with more than two levels. Perhaps a major limitation of this study was the use of cross-sectional data, which restricts our ability to establish temporal relationships between predictors and stunting. The predictors (for instance socio-economic status) and the outcome (stunting) were collected at the same time, it was not possible to determine if the exposures preceded the outcome. Due to the cross-sectional nature of the data that we used, the model we have produced is a diagnostic prediction model to identify children their current stunting status. However, it could be used on children who are not yet stunted to calculate their risk of being stunted in the future. Future research with prospective cohort designs is needed to develop accurate prognostic models for stunting. The study did not consider least-angle regression (LARS) due to its complexity in implementation in R as another variable selection method for nonlinear models as another variable selection method for nonlinear models, which is considered a limitation of this study. Another limitation is that the study assumed a logistic link function provides a better predictive model. However, the study could have used a probit link function or complementary log–log link function. Lastly, the literature search was limited to PubMed and Google Scholar, while other databases such as Web of Science and Scopus could have been utilized and would have provided additional studies on child stunting in Sub-Saharan Africa.

## Conclusion

The study has shown the viability of deriving child stunting diagnostic models that could be used to assess current child stunting status. Timely identification of children that are more likely to be stunted may help prevent the future impact of stunting and alleviate the disease burden in low-resource settings.

### Supplementary Information

Supplementary Material 1.

## Data Availability

The data generated and analysed from this study are available upon request from the corresponding author. The original 2015/16 Malawi DHS data set is available on the DHS Program website: https://dhsprogram.com/.

## Appendix

### Additional sources

[47–65]
